# Energy flow analysis of laboratory scale lithium-ion battery cell production

**DOI:** 10.1016/j.isci.2021.102437

**Published:** 2021-04-16

**Authors:** Merve Erakca, Manuel Baumann, Werner Bauer, Lea de Biasi, Janna Hofmann, Benjamin Bold, Marcel Weil

**Affiliations:** 1 ITAS, Institute for Technology Assessment and Systems Analysis, KIT, Karlsruhe, Germany; 2 HIU, Helmholtz-Institute for Electrochemical Energy Storage, KIT, Ulm, Germany; 3 CICS.NOVA - OAT, Universidade NOVA de Lisboa, Campolide, Lisbon, Portugal; 4 IAM – ESS, Institute for Applied Materials - Energy Storage Systems, KIT, Karlsruhe, Germany; 5 wbk, Institute of Production Science, KIT, Karlsruhe, Germany

**Keywords:** Electrochemical Energy Storage, Energy Resources, Energy Engineering, Manufacturing

## Abstract

Lithium-ion batteries (LIBs) have been proven as an enabling technology for consumer electronics, electro mobility, and stationary storage systems, and the steadily increasing demand for LIBs raises new challenges regarding their sustainability. The rising demand for comprehensive assessments of this technology's environmental impacts requires the identification of energy and materials consumed for its production, on lab to industrial scale. There are no studies available that provide a detailed picture of lab scale cell production, and only a few studies provide detailed analysis of the actual consumption, with large deviations. Thus, the present work provides an analysis of the energy flows for the production of an LIB cell. The analyzed energy requirements of individual production steps were determined by measurements conducted on a laboratory scale lithium-ion cell production and displayed in a transparent and traceable manner. For the comparison with literature values a distinction is made between the different production scales.

## Highlights

• Energy analysis of lab scale lithium-ion pouch cell production
• The energy data stem from in-house electricity measurements (primary data)
• The main contributors are coating, drying, formation process, and the dry room
• Different production scales such as lab, pilot, and industrial are compared

## Graphical abstract

**Figure:**
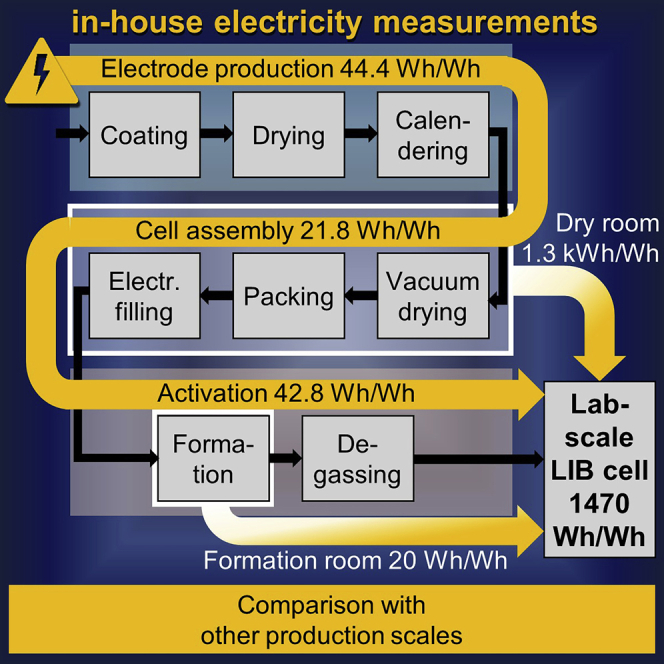

## Introduction

Since their commercialization in 1991, the worldwide demand for lithium-ion batteries (LIBs) has steadily increased (Blomgren, 2017; Vaalma et al., 2018). They are the main factor in the success of consumer electronics, electro mobility, and stationary storage systems. In addition, policy requirements and market adaptation to more environmentally sustainable products have contributed to the increasing demand for electric vehicles and consequently for batteries (McManus, 2012; Thomitzek et al., 2019b). This widespread application of LIBs has led to important advances (Peters et al., 2017). However, with the increasing demand for batteries, new challenges arise regarding their competitiveness and sustainability (Thomitzek et al., 2019b). There is a strong need to prospectively identify non-intended effects as far as possible, e.g., potential negative environmental impacts, rather than to tackle them when they become apparent after the technology enters the market. It is crucial to provide a broad picture about the early development stage of a technology, beginning with lab scale processes by addressing potential innovation obstacles or unintended impacts as early as possible (Arvidsson et al., 2018; Thonemann et al., 2020; Thonemann and Schulte, 2019).

It is clear that reducing the energy required for the production of a battery (or any other technical device) would have a positive effect on its environmental sustainability (Thomitzek et al., 2019a, 2019b). Yet this requires detailed knowledge of the energy demand of LIB production ranging from a lab to industrial scale. The industrial scale has been discussed in several studies (Davidsson Kurland, 2019; Ellingsen et al., 2014; Peters et al., 2017; Thomitzek et al, 2019a, 2019b; Yuan et al., 2017), most recently in the review by Emilsson and Dahllöf (2019). However, the production of LIBs is very complex, and access to data from industrial manufacturers is limited (Dai et al., 2019; Davidsson Kurland, 2019; Ellingsen et al., 2014; Peters et al., 2017; Yuan et al., 2017). The availability of such data is particularly important for conducting life cycle assessments (LCAs), which are a well-established, standardized method for evaluating the environmental impacts of products and goods but also activities (Peters et al., 2016; Thomitzek et al., 2019b; Zackrisson et al., 2010). Potential environmental impacts of LIBs have been analyzed several times in literature (Dai et al., 2019; Dunn et al., 2012; Ellingsen et al., 2014; Emilsson and Dahllöf, 2019; Majeau-Bettez et al., 2011; McManus, 2012; Notter et al., 2010; Peters et al., 2017; Zackrisson et al., 2010). Most studies are based on secondary data or rough estimations and have a low level of transparency (Ellingsen et al., 2014; Peters et al., 2017; Schönemann, 2017; Thomitzek et al., 2019b; Yuan et al., 2017). The batteries examined in the literature vary greatly in their characteristics, such as size, storage capacity, lifetime, and cell type. Furthermore, the studies have different scope and system boundaries and different assumptions for certain parameters and production processes (Peters et al., 2017; Thomitzek et al., 2019a). In addition, only a few studies provide detailed and comprehensible information about battery cell production, which is repeatedly declared to be the production step with the highest energy demand and a high environmental impact (Dai et al., 2019; Davidsson Kurland, 2019; Emilsson and Dahllöf, 2019; Yuan et al., 2017). Thus, the results of studies on the environmental impact of an LIB differ considerably, and some studies rely on outdated data (Davidsson Kurland, 2019; Peters et al., 2017). Finally, a low number of available studies take a detailed look at lab scale production of battery cells and the potential implications for upscaling, which would allow the identification and prioritization of important technology properties and provide a broader basis for both decision-making and “early warning” in early stage technology development. Hence, there is a need for studies that investigate the energy demand related to LIB production based not only on primary data, but which also reveal a detailed allocation of the energy demand to the analyzed production steps, especially related to early stage technology development. This requires high transparency regarding the battery characteristics, the system boundaries, and assumptions, as this is key to assuring not only comprehensibility but also comparability of the results.

The aim of this study was to conduct a bottom-up analysis of the energy flows of an LIB cell production based on reference processes at the Battery Technical Center (BTC) of the Karlsruhe Institute of Technology (KIT), which consider different cell manufacturing levels. Existing literature contains little distinction between the energy demand according to the production volume. This study fills this gap, not only by providing values for a lab scale production but also by providing first analyses of the values already published in the literature in relation to their production capacity. This provides a robust foundation for future early technology-development-oriented sustainability assessments in the field of, e.g., LCA. Each manufacturing step and related hot spots in production are identified, compared, and discussed based on our measurements and the most recent literature, to provide corresponding bandwidths. Here, a distinction is made between literature from the LCA field and literature handling explicitly the energy demand of cell manufacturing.

In this study, cell production was carried out on a laboratory and medium scale industry level and assembled within a semi-automated manual production line in a dry room. Although some production steps at the BTC are available on a medium or pilot scale, the term laboratory scale production is used here. It should be noted that process steps on this level are performed manually and at a lower standardization degree compared with industrial processes: a scale-up in combination with a comprehensive literature review is carried out to explore the transferability of the results to large-scale LIB production processes. This approach is also highly relevant for technologies with low technology readiness levels (TRL), which are currently only developed and produced on a laboratory scale such as sodium-ion batteries (SIBs) (Peters et al., 2016). Using laboratory scale data, prospective LCAs can be generated for technologies with low TRL. Energy requirements and critical materials, and consequently potential environmental impacts, can thus be determined prior to their industrial scale production.

The article is structured as follows: “Methodology” outlines the material and energy flow analysis and the general approach of this work. The production of an LIB cell is described, and a comprehensive literature review on the energy requirements of an LIB production is examined in “Background Information” section. In “System Boundaries and Assumptions” the consideration of the production steps and the framework conditions, simplifications, and calculations are discussed. Section “Results” starts with an overview of the results, firstly for the individual production steps and secondly for the spatial environment. These results are then compared with the values obtained from the literature. The final results are analyzed in “Discussion and Sensitivity Analysis,” with a “Conclusion and Outlook” in the following section. Additional information can be found in the supplemental information.

### Methodology

This work borrows some major principles from material and energy flow analysis (MEFA) (Teresatorres et al., 2008), which can be considered as a modification of the classical material flow analysis (MFA). This method is also highly relevant for LCAs (DIN EN ISO 14044, 2006). An MFA investigates incoming materials to a given system, the flows inside that system, and the related outputs of that system to other systems (Hendriks et al., 2000). Based on the conservation of materials the results can be controlled by a simple material balance, where all inputs and stocks of a process must be in equilibrium with its outputs (Ayres and Ayres, 2002; Brunner and Rechberger, 2004; Fischer-Kowalski, 1998). This study follows the principles of a classical MFA approach as described by Hendriks et al. (2000) (according to Baccini and Brunner (1991), Brunner et al. (1990) and Baccini and Bader (1996)) with a focus on energy flows:

1. Determination of the research question and goal definition.
2. Description of the system by means of system boundaries and selection of relevant processes.
3. Data collection by measurements, market analysis, and expert interviews.
4. Modeling and balancing of incoming and outgoing flows.
5. Interpretation of results.

**Figure 1:**
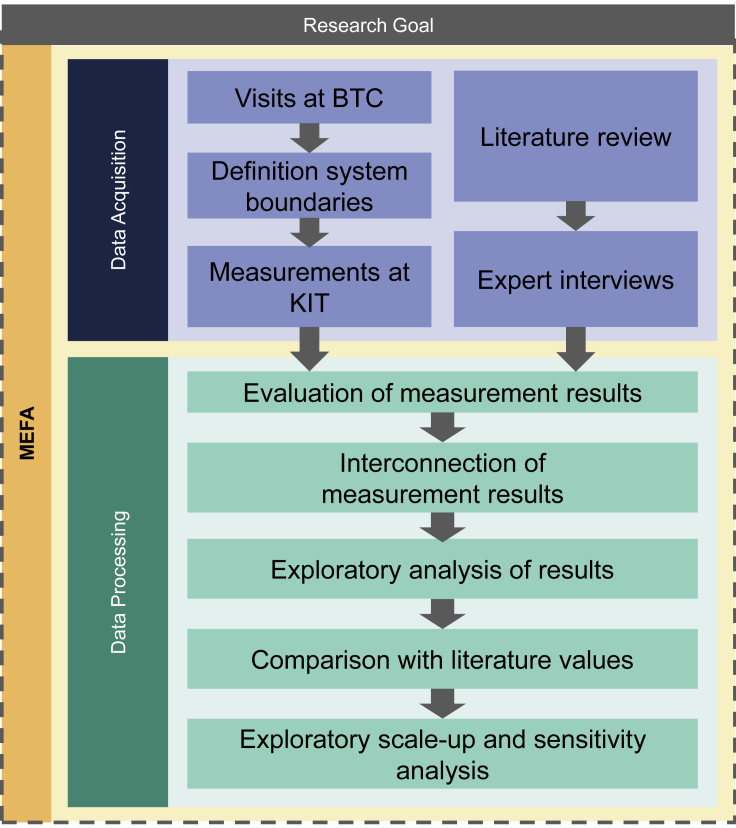
General MEFA-approach of this work

As indicated in Figure 1, several visits to a manufacturing line at KIT were conducted to get an overview of the production steps available and to define the system boundaries of this work. The data obtained by measurements are supplemented with expert interviews. Specifically, a plant engineering company that manufactures dry rooms and an expert for battery technologies were interviewed in the beginning of this study. Latter was interviewed regarding the production step coating and drying. At the same time, experts for lab and pilot scale LIB cell production of the BTC were consulted.

It is clear that energy consumption of processing machines varies, depending on the size, make, model of the machine, and the operating parameters (temperature, speed, full load, or partial load). Thus, all the data related to machinery, and the measurement process itself, are described in detail in the supplemental information, to which we refer where appropriate to ensure the comprehensibility and transparency of this work.

A comprehensive literature review determines existing values for the energy requirements of an LIB production. The findings obtained through the literature review are compared and analyzed with the results of this work. Because this work investigates production on a laboratory scale, a scale-up is performed specifically for the dry room in order to provide a tendency for the energy consumption at larger production scales. The functional unit (FU) of this work is Wh per Wh cell energy storage capacity.

The energy data are gathered by conducting measurements for each process step to provide detailed primary data. This distinguishes this work from previous studies and highlights the benefits of the results. For more detailed information on the measuring device, please refer to chapter 2 Measuring Device, in the supplemental information.

All the processes prior to the mixing of the slurry, e.g. raw material extraction, and downstream processes, such as assembling the cell into a module or recycling processes, are not considered here, but can be found, e.g., in Peters et al. (2019).

### Background information

In the following, relevant background knowledge is presented for a better understanding of this work. First, a description of the production of an LIB is given. Secondly, the literature review findings on the energy demand of LIBs that serve as a basis for this work are discussed.

#### Production of a lithium-ion battery cell

**Figure 2:**
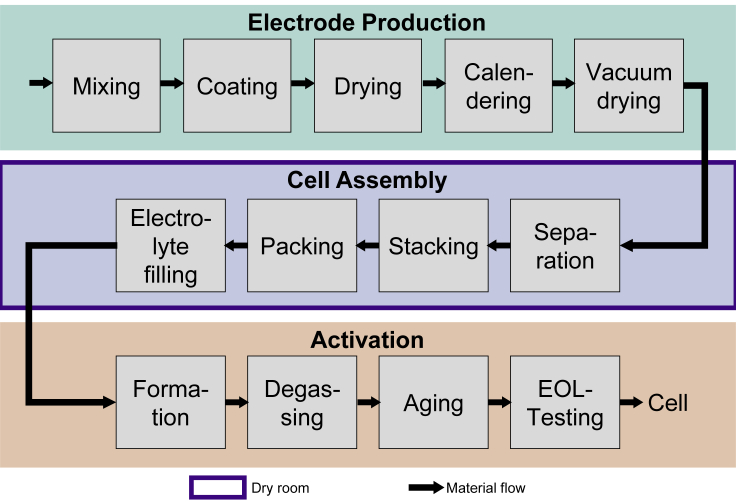
Schematic illustration of the production steps of pouch cells

In general, the production of an LIB cell can be divided into three main steps: the electrode production, the cell assembly, and the activation of the cell (Heimes et al., 2018; Kampker, 2014; Korthauer, 2018; Kurzweil and Dietlmeier, 2018). Figure 2 illustrates the production of a pouch cell schematically based on Kampker (2014), as described below.

In electrode production, the slurry is mixed and applied to the carrier foil (aluminum for cathode and copper for anode) (Heimes et al., 2018; Kampker, 2014; Korthauer, 2018; Kurzweil and Dietlmeier, 2018). The coated film is dried in a continuous process (Heimes et al., 2018; Kampker, 2014). Thereby the solvent is removed from the material by heat. Once dried, the electrode film is calendered, meaning it is compressed by a pair of rotating rollers (Heimes et al., 2018; Kampker, 2014; Korthauer, 2018). The calendered electrode is transported to the vacuum drier and stored for up to 30 h, where residual moisture and solvent are removed from the electrode by evaporation at temperatures above 100°C. Afterward the dried coils are brought into the dry room and the cell assembly begins (Heimes et al., 2018; Kurzweil and Dietlmeier, 2018). Due to the high reactivity of the electrolyte (LiPF_6_) with moisture, cell assembly, especially electrolyte filling, has to be performed in an extremely dry atmosphere (Korthauer, 2018). Separation is the first step in cell assembly. It involves cutting individual anode and cathode sheets out of the electrode tapes and sometimes also separator sheets. Metal contact tabs, which take over the current transport out of the cell, are welded with ultrasound to the electrode sheets. Anode, cathode, and separator have to be rolled or stacked to produce either a cylindrical or brick-shaped cell body. Only the stacking process is considered here, as it is usually found in a laboratory environment. The cell stack is therefore placed in a deep-drawn plastic aluminum composite pouch foil and sealed gas-tight. Because the electrolyte has to be filled in the next step, one side remains unsealed (Heimes et al., 2018; Kampker, 2014; Kurzweil and Dietlmeier, 2018). After electrolyte filling, the pouch cell is evacuated and sealing is completed. The activation of the cell represents the final main step of the lithium-ion cell production and begins with the formation of the cell. Here, the cell is charged and discharged for the first time, and the Solid Electrolyte Interface (SEI) is formed (Heimes et al., 2018; Kampker, 2014; Korthauer, 2018). During the formation of pouch cells, the first charging process is responsible for the generation of gas, which is captured in the gas pocket. Therefore, the next steps involve the degassing. Following this, cell aging is performed. Aging involves the maturation of the cells and secures their quality. End-of-line testing is the final step of LIB cell production (Heimes et al., 2018; Kampker, 2014).

#### Literature review

A literature review (see Table 1) was conducted to provide an overview of existing values for the energy demand of different levels of LIB cell production (laboratory, pilot, and industrial scale). The studies selected deal with the energy requirements or the environmental impacts for the production of an LIB. A distinction between the levels of production from lab to pilot up to industrial scale is made. In addition, a differentiation has been made between studies stemming from the LCA field, and those explicitly focusing on identifying the energy demand of battery manufacture. This is indicated in Table 1 in order to derive any significant differences among these types of studies. Only studies published after 2010 are included.

**Table 1:** Studies with a focus on LIB production published after 2010

Year	Author	Cell Type	Cell Voltage [V]	Cell Capacity [Ah]	Specific Energy [Wh/kg]	Single Cell Weight [kg]	Active Material Cathode	Active Material Anode	Solvent	FU	Energy Demand [kWh]	Dry Room	Scale	Data Source p = Primary; s = Secondary	LCA Related
2019	Dai et al.	Prismatic	n/a	46	197 (cell)	0.8555^a^	NMC111	Graphite	NMP	per kWh cell	47^b^	Yes	Industrial	p and s from industry partner	Yes
2019a	Thomitzek et al.	n/a	33		n/a	n/a	n/a	n/a	NMP	per kWh cell	744.6	Yes	Pilot	p from own facility	No
2019b	Thomitzek et al.	n/a	33.3		121.53^a^ (cell)	0.274	NMC622	Graphite	NMP	per kWh cell	1150	Yes	Pilot	p from own facility	No
2017	Pettinger and Dong	n/a	3.7	20.5	200 (battery)	0.45	n/a	n/a	NMP	per cell	3.306	No	Industrial	p from industry partner	No
2017	Yuan et al.	Pouch	3.85	32	141.94^a^ (cell)	0.868	LMO	Graphite	NMP	per cell	13.28	Yes	Industrial Pilot	p measured from pilot scale industry partner	No
2016	Kim et al.	Pouch	3.7	n/a	140 (cell)	0.391^a^	LMO/NMC	Graphite	NMP	per kg battery	33.33^a^	Yes	Industrial	p from industry partner	Yes
2015	Dunn et al.	n/a	n/a	n/a	n/a	n/a	LMO,LCO, LFP, NMC, LMR-NMC	Graphite	NMP	per kg battery	for NMC: 4.5 to 780	Yes	Industrial	s	Yes
2014	Ellingsen et al.	Pouch	3.65	20	n/a	n/a	Li(Ni_x_Co_y_Mn_z_)O_2_	Graphite	NMP	per kWh battery cell capacity produced	Average: 643.89 Energy-efficient: 162.78	Yes	Industrial	p from industry partner	Yes
2014	Li et al.	Prismatic/Pouch	3.65	27	n/a	n/a	NMC111	SiNW	n/a	per kg cell	unclear	n/a	Laboratory	s	Yes
2012	McManus	n/a	n/a	n/a	128 to 200 (battery)	n/a	n/a	n/a	NMP	per kg battery	25^c^	n/a	n/a	s	Yes
2011	Majeau-Bettez et al.	n/a	3.7	n/a	140 (cell)	n/a	NMC	Graphite	NMP	per kWh battery capacity	0.33^a^	n/a	n/a	s	Yes
2010	Notter et al.	Prismatic	n/a	n/a	114 (battery)	n/a	LiMn_2_O_4_	Graphite	n/a	per kg cell	0.124^d^	n/a	n/a	s	Yes
2010	Zackrisson et al.	n/a	n/a	n/a	93 (battery)	0.967	LiFePO_4_	Graphite	NMP	per kg battery	20.5^e^	n/a	n/a	s	Yes

a Value calculated according to data obtained from paper and/or corresponding supplemental information.

b 8 kWh electricity, 39 kWh steam.

c Cumulative energy demand for a battery.

d 0.106 kWh electricity, 0.018 kWh process heat.

e 11.7 kWh electricity, 8.8 kWh gas.

The main findings of the literature review can be summarized as follows:

• Table 1 reveals data gaps in the literature and also indicates large differences between the results of the examined studies.
• The reason for this is the different battery technologies, assumptions, system boundaries, and simplifications that are used for the determination of the energy requirements; this is in line with the findings of the review by Peters et al. (2017).
• Widely ranging production scales were examined in the studies, although this has a significant impact on energy requirements.
• Although there are several studies of industrial and pilot scale LIB production, there is only one study on the energy demand of laboratory scale LIB production.

These findings are discussed in more detail below.

As Ellingsen et al. (2014) emphasized, there is a lack of transparency in the provided data in previous studies. Table 1 underlines this criticism by showing that only 6 of the 13 examined studies reveal the cell geometry, which can be decisive for the energy requirement, because the production of a pouch cell differs from that of a cylindrical or prismatic cell. For example, Li et al. (2014) mention that a prismatic cell is examined, but in the corresponding supplemental information the energy requirement for a pouch cell is itemized. This makes it difficult to understand the cell type the energy demand is based on. The cell materials are also decisive for the resulting energy demand. For example, if water instead of NMP is used, less energy is required for drying the electrodes (Pettinger and Dong, 2017). However, hardly any data on the solvent are available in the work of Notter et al. (2010) and Li et al. (2014). Further, different cathode and anode materials are used in the studies (see Table 1). In the cases of Pettinger and Dong (2017) and McManus (2012) there is hardly any data regarding the active materials. Li et al. (2014), however, investigate anodes with silicon nanowires, in contrast with the other studies, which use graphite. Moreover, some studies (Dunn et al., 2015; Ellingsen et al., 2014; Li et al., 2014; Majeau-Bettez et al., 2011; McManus, 2012; Notter et al., 2010) lack data on cell characteristics such as cell voltage, cell capacity, specific energy, or single cell weight. These data would be important in order to translate the different FUs of the studies into a single unit, because this is necessary for the comparison of the reported values and also to identify the source of differences or similarities in the results.

Ellingsen et al. (2014) mentioned the extremely high share of the dry room in the overall energy demand of battery production. This corresponds well with the findings of Emilsson and Dahllöf (2019), which noted in their review that the energy requirements for the dry room have been underestimated or even neglected in earlier studies. Pettinger and Dong (2017) investigate a cell production process mainly without the use of a dry room. Li et al. (2014), McManus (2012), Majeau-Bettez et al. (2011), Notter et al. (2010), and Zackrisson et al. (2010) do not provide any exact information on whether the dry room was taken into account or not. In addition, Table 1 reveals that most of the studies with LCA reference mainly use secondary data (from previous studies or LCA databases) (Dai et al., 2019; Dunn et al., 2015; Li et al., 2014; Majeau-Bettez et al., 2011; McManus, 2012; Notter et al., 2010; Zackrisson et al., 2010), which poses a high risk for inaccurate values, as well as the spreading of failures, or the use of outdated data. This interdependence of the different LCA studies on LIBs has previously been discussed in detail by Peters et al. (2017). Some of the studies mainly focus on entire battery pack production and not on cell production, in particular Kim et al. (2016), Dunn et al. (2015), McManus (2012), Majeau-Bettez et al. (2011), and Zackrisson et al. (2010); the reported energy demand here is consequently also related to the entire battery pack rather than the cell manufacturing process.

The production size can be decisive because an industrial scale production requires less energy per cell than a pilot or laboratory scale production (Li et al., 2014). This stems especially from the different production rates. Five of the investigated studies refer to an industrial cell production (Dai et al., 2019; Dunn et al., 2015; Ellingsen et al., 2014; Kim et al., 2016; Pettinger and Dong, 2017), two to a pilot scale production (Thomitzek et al, 2019a, 2019b) and one to data from a laboratory scale production (Li et al., 2014). Four of the studies do not give explicit information about the production volume (Majeau-Bettez et al., 2011; McManus, 2012; Notter et al., 2010; Zackrisson et al., 2010). It is striking that although Yuan et al. (2017) report a pilot scale production, they use less energy than the most energy-efficient value of Ellingsen et al. (2014). Davidsson Kurland, 2019 estimated the energy demand for two Gigafactories, Northvolt (Sweden) and Tesla (USA), and reported 50 kWh per kWh battery cell manufacturing capacity for Northvolt and 65 kWh per kWh battery cell manufacturing capacity for Tesla. These newer values of modern, industrial battery manufacturers are similar to the values of Dai et al. (2019) and Pettinger and Dong (2017). However, the studies of Dai et al. (2019), Notter et al. (2010) and Zackrisson et al. (2010) name not only electricity but also gas, steam, or process heat as an energy source. In particular, the revealed energy demand of Dai et al. (2019) consists of 83% steam. The only study providing energy requirements for a laboratory scale production is based on anodes with silicon nanowires instead of graphite (Li et al., 2014), making it difficult to compare the other studies investigating graphite anodes. Thus, it is hardly possible to analyze the scale-up effect of the laboratory production investigated by Li et al. (2014) to pilot or industrial production scales.

Overall, this literature review underlines the aforementioned lack of transparency and the lack of dependability of the data mentioned in the studies. Given the stated conditions, a reliable comparison of the results of most studies is therefore hardly possible. Moreover, the only lab scale study is outdated, as it was published in 2014 and uses anodes with silicon nanowires (Li et al., 2014). Hence, there is a lack of lab scale studies on the energy demand of LIB cell production on a laboratory level. However, due to the increased demand for batteries, the focus on battery cell production is rising (Emilsson and Dahllöf, 2019), and more detailed data are provided in studies without LCA references by Thomitzek et al. (2019a), Pettinger and Dong (2017), and Yuan et al. (2017). Due to the more extensive information provided on the individual manufacturing processes, these studies are analyzed in more detail below.

Thomitzek et al. (2019a) performed an energy and material flow analysis on a research character battery production of the pilot scale Battery LabFactory Braunschweig. Pettinger and Dong (2017) investigated a large-scale operation line of the battery manufacturer SOVEMA. Yuan et al. (2017) provide a detailed analysis of the energy requirements for the production of lithium-ion batteries at the Johnson Controls pilot plant. Unlike the remaining studies (Dai et al., 2019; Dunn et al., 2015; Ellingsen et al., 2014; Kim et al., 2016; Li et al., 2014; Majeau-Bettez et al., 2011; McManus, 2012; Notter et al., 2010; Thomitzek et al., 2019b; Zackrisson et al., 2010) from Table 1, they reveal a detailed breakdown of the energy demand according to the individual process steps, as displayed in Table 2. Therefore, the next step is a detailed analysis of these three studies.

**Table 2:** Energy demand in Wh per Wh cell energy storage capacity for processes from selected studies inspired by Thomitzek et al. (2019a)

	Thomitzek et al., 2019a (Pilot Scale)	Pettinger and Dong, 2017 (Industrial Scale)	Yuan et al., 2017 (Pilot Scale)	Range x_max_-x_min_
	[Wh per Wh cell energy]	[Wh per Wh cell energy]	[Wh per Wh cell energy]	[Wh]
Mixing	10.5	2.6^f^	0.9	9.6
Coating and drying	133.6	15.4	51.0^h^	118.2
Calendering	20.7	5.9	3.0	17.7
Separation	0.1	5.2^g^	5.7^i^	5.6
Stacking and packing	1.9^a^,^b^	6.0	9.0^j^,^d^	7.1
Vacuum drying	6.0^c^	6.0	n/a	0.0
Electrolyte filling	8.7^d^	1.5	4.7	7.2
Formation	26.1	2.8	0.6^k^	25.5
Aging	87.7	n/a	n/a	n/a
Dry room	448.7^e^	n/a	31.2	417.5
Others	0.6	0.4	n/a	0.2
**Total**	**744.6**	**45.9**	**106.2**	**698.7**

a Originally named as packaging.

b Contacting, housing, and deep-drawing summarized.

c Originally named as final drying.

d Including final sealing.

e Originally named as technical building services.

f Originally named as slurry preparation.

g Slitting and notching summarized.

h Coating and drying summarized.

i Originally named as notching.

j Originally named as welding.

k Originally named as pre-charging.

In order to enable a comparison of the results, individual process steps are partially summarized into a broader category. The exact division can be found in notes below Table 2. The column “Range” shows the difference between the highest and the lowest energy demand. Remarkably, the highest energy consumption is most often drawn from the pilot scale production of Thomitzek et al. (2019a). Pettinger and Dong (2017), by contrast, tend to have lower energy requirements for production on an industrial scale and have no maximum value in any category.

All three studies show that due to the high drying temperature, coating is the production step with the highest energy demand, but with large deviations between the reported values. Although Thomitzek et al. (2019a) give the highest value with 133.6 Wh per Wh cell energy storage capacity, the energy requirement of Pettinger and Dong (2017) with 15.4 Wh per Wh cell energy storage capacity is only about 11.5% of this. According to the analyzed literature, a significant difference exists between the energy requirements for the dry room. Thomitzek et al. (2019a) distinguish a 14.4 times higher energy demand than Yuan et al. (2017). Pettinger and Dong (2017) did not report any energy demand for the dry room. However, this has a significant impact on the energy demand. If the dry room is not considered, the demand is only 295.9 Wh per Wh cell energy storage capacity in Thomitzek et al. (2019a) and 75 Wh per Wh cell energy storage capacity in Yuan et al. (2017). Another difference between the data presented in the studies is aging, which is only considered in the study of Thomitzek et al. (2019a). At 87.7 Wh per Wh cell energy storage capacity, this process is responsible for 11.6% of the total demand in Thomitzek et al. (2019a).

Overall, clear differences can be seen in the energy requirements of the individual processes as shown in Table 2, making it difficult to provide generalizable information for battery cell production processes based on these values. This is partly due to the fact that Thomitzek et al. (2019a) and Yuan et al. (2017) analyzed a pilot scale production, whereas Pettinger and Dong (2017) investigated an industrial scale production. Secondly, there are differences in the properties of the cells studied, such as the cell geometry, which has a decisive influence on the energy demand but remains unknown in the work of Thomitzek et al. (2019a).

### System boundaries and assumptions

The LIBs manufactured at the KIT, especially at the BTC, are mainly pouch cells. Thus, this work is dedicated to the energy and material flows of a pouch cell. The analyzed battery is a “KIT 20” cell with a rated capacity of 20 Ah, a nominal voltage of 3.7 V, and a gravimetric energy density of 141 Wh∙kg^−1^. One cell weighs 540 g and has the dimensions of 179 mm × 236 mm x 7.4 mm (KIT 20 Li-ion cell, 2020). The cathode active material is NMC111, and the anode active material is graphite. Because the cathode sheets of the KIT 20 cell are purchased components, NMC622 cathodes were used for the measurements while coating and drying. The used electrolyte is 1 M LiPF_6_ in EC/DMC (1:1) wt % + 3% VC. Because the separator is a purchased part, no measurements are performed on its manufacturing. All determined values are set in relation to the KIT 20 cell and are calculated based on a fully utilized production at KIT. A picture and a detailed illustration of the KIT 20 pouch cell and its dimensions can be found in Figures S1 and S2 in chapter 1 KIT 20 pouch cell in the supplemental information.

**Figure 3:**
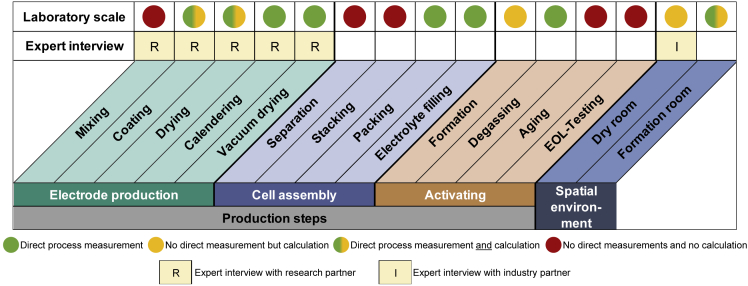
Consideration and connection of production steps

The energy demand of the production steps of cell manufacturing taken into account and measured are shown in Figure 3. The production steps marked with green dots are those that were measured and thus provide primary data. No direct measurements are carried out for the production steps marked with yellow dots; these are calculated using other available data, such as the load curves of the entire building for the energy demand of the dry room. This represents a limitation of the assessment but is considered as sufficient to provide a robust indication of the energy requirements. The green and yellow dotted process steps are analyzed using both primary data by direct measurements and data obtained by corresponding calculations, as shown in chapter 3.9 in the supplemental information. However, due to the laboratory character of the production, some steps are performed manually. Therefore, no measurements and calculations are possible for the production steps marked with red dots. Furthermore, expert interviews with internal and external research partners are conducted for the electrode production. In addition, an expert interview is carried out with an industrial partner for the dry room.

Although not covering all production steps of a cell manufacturing process, the key steps such as coating and drying of the electrode as well as the formation of the cell are included. Because this work considers cell production on a laboratory scale, all steps required for this production are covered. In addition, the energy requirements for the dry room and the air conditioning of the formation room are taken into account. In addition, the energy demand for preheating or system start-up was recorded in all appropriate steps (e.g. coating and drying) and distributed among the manufactured cells. Further information on this topic can be found in the supplemental information.

It should be noted that the production is not a continuous process. The machines are sited in different locations. The data collection and production were not chronological but rather staggered over time. Several separate cell production campaigns were run to be able to carry out the measurements. Overall, the measurements were performed over a period of approximately 2 weeks. First, the process steps of vacuum drying, ultrasonic welding, sealing, electrolyte filling, formation, degassing, and final sealing were measured. These measurements were conducted within 1 week. In the following week the measurements for coating, drying, and calendering of the electrodes were carried out. Consequently, purchased rather than measured electrodes were used in the cell assembly. The exact process parameters and framework conditions for the measurements (e.g. different temperatures, speeds, and types of load) can be found in chapter 3 Measuring Procedure in the supplemental information.

For the production steps labeled with green dots in Figure 3, measurements are carried out at the respective machines. Different speeds are measured for coating and drying the electrode. The average speed is 0.36 m∙min^−1^. Drying is carried out in a two-chamber system, with a temperature of 80°C in the first chamber and 120°C in the second. The measurement is performed on a cathode. Different speeds are also used for calendering in order to determine their impact on the energy demand. The average calendering speed is 3 m∙min^−1^ at a temperature of 50°C. The exact results and parameters can be found in chapter 3.1 to 3.7 of the supplemental information. For the cell assembly, two identical cells are manufactured, and the average energy consumption of both cells is used. In addition, the energy requirement for the air conditioning system of the formation room is measured, as described in more detail in chapter 3.8 Formation Room in the supplemental information.

For the processes marked with yellow dots in Figure 3 no measurements are possible. The values for those steps are based on calculations. Because only the production of a cathode is measured, the energy requirement for coating and drying of the anode is determined based on calculations. It is assumed that the energy requirement of the anode is about 15% less than for the process of the cathode (Pettinger, 2019). It is also not possible to perform measurements for the energy demand of the dry room. However, as mentioned repeatedly in the literature, the dry room contributes significantly to the energy consumed in LIB cell production (Ellingsen et al., 2014; Emilsson and Dahllöf, 2019). To be able to provide a reliable energy demand for the dry room, the value is determined based on calculations. For this, the load values for the month of May 2019 of the entire building are identified. Then the difference between the days with a switched-on or switched-off dry room are calculated, leading to an operating power of 64.8 kW for a surface area of 100 m^2^. Thereby, the dry room ambient temperature is 22°C and provides a dew point temperature of −70°C. For more detailed information, please refer to chapter 3.9 Dry Room in the supplemental information. The formation of the cell is also determined by calculation. For this purpose, the supplied energy of the three-stage cyclization process is added to the energy requirement of the cycler itself. A more detailed overview of the conducted calculations can be found in chapter 3.6 Formation in the supplemental information.

## Results

In the following, the results of the measurements are presented. First, the results of the individual production steps are displayed, supplemented by the energy requirements for the spatial environment. Then, the results obtained from this work are compared with the values available in the literature.

### Processes and spatial environment

**Figure 4:**
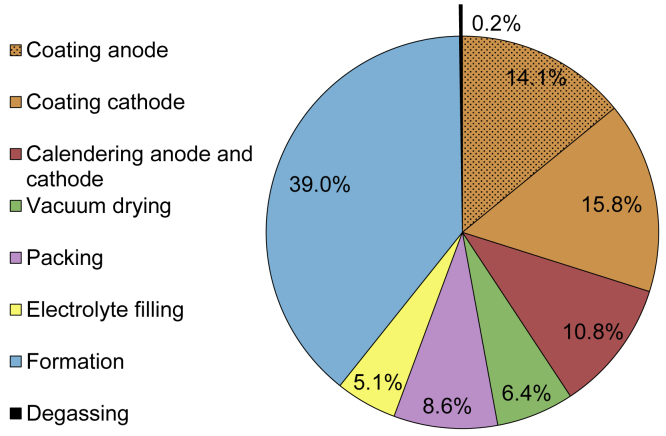
Relative distribution of the total energy requirement for all production steps without spatial environment

The total energy requirement for the production steps without the spatial environment (dry and formation room) of a cell is 8.3 kWh, which equals an energy demand of 109.01 Wh per Wh cell energy storage capacity. As can be seen in Figure 4, which displays the energy demand of the production steps without the spatial environment, the highest energy requirements are for coating with a total of 29.9%, calendering with 10.8%, and formation with 39.0%. Overall, these three process steps on their own are responsible for 79.9% of the energy consumption. The remaining processes of vacuum drying, packaging, electrolyte filling, and degassing have an overall energy requirement of 20.1%.

The total energy requirement for the spatial environment is 1360.39 Wh per Wh cell capacity produced. With 1339.64 Wh the dry room requires significantly more (factor 65) energy compared with the formation room. The determined value for the dry room indicates an extremely high energy demand, which is analyzed in more detail in section 6. In conclusion, the total energy demand for the production of a cell on a laboratory scale equals 1469.53 Wh per Wh cell energy storage capacity, as displayed in Table 3.

**Table 3:** Total energy demand for laboratory LIB cell production in Wh per Wh cell energy storage capacity

Energy Demand in	Electrode Production		Cell Assembly			Activation		Spatial Environment		Total
	Coating	Calendering	Vacuum Drying	Packing	Electrolyte Filling	Formation	Degassing	Dry Room	Formation Room	
**Wh per Wh cell**	32.57	11.82	6.96	9.32	5.52	42.55	0.26	1339.64	20.75	**1469.53**
**%**	2.2	0.8	0.5	0.6	0.4	2.9	0.0	91.2	1.4	**100**

### Comparison with other studies

**Figure 5:**
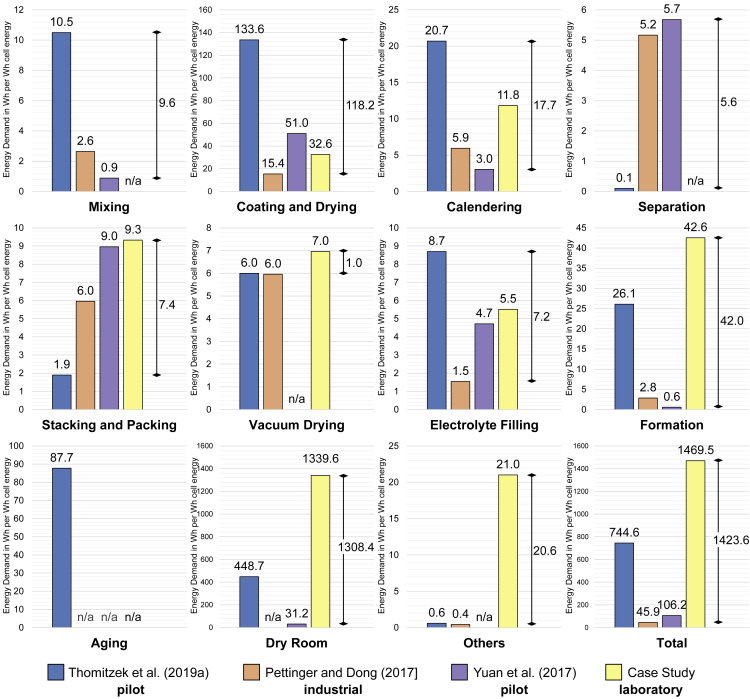
Energy demand in Wh per Wh cell energy for LIB cell production in comparison with other studies

In the following, the results of this study are compared with the values available in the literature. For this purpose, the studies mentioned in Table 2 are used, as they provide an exact breakdown of the energy requirements for the individual process steps, as well as type of production (lab, pilot and industrial scale), and thus enable statements regarding similarities and discrepancies. For better comparability, the values from Table 2 are combined and compared with the determined values of this study in a bar chart, as displayed in Figure 5. Figure 5 also shows the range between the highest and lowest value for each production step on the right.

As Figure 5 indicates, some process steps in this work, but also in the other studies, are partly missing. However, the values available in the literature demonstrate that the missing process steps of this work (mixing and separation) do not have a considerable influence on the energy demand. In contrast, the process steps with a massive influence, such as coating and drying, or the dry room, are covered, underlining the robustness and reliability of the results of this work. It can be said that overall, the lowest energy demand is provided by the industrial scale production of Pettinger and Dong (2017) and the highest by the lab scale production within this study. However, when looking at the individual results at process level this is not always the case. It is not possible to identify specific sources of such deviations within this work, as the exact operating conditions of the investigated studies are not available. Thus, further research is required in order to identify the source of some of the conflicting trends at process level. Concrete information on the operation of the processes such as the machines, their parameters, and the spatial conditions is required. In the following, the main differences shown in Figure 5 are discussed.

The most considerable difference in the results can be found for the dry room. Here, the difficulty in the comparability of the studies is once again evident, because some production steps and parameters are missing, as mentioned above. With 1339.64 Wh, the value of this study is three times higher than Thomitzek et al. (2019a) and 42 times higher than the result of Yuan et al. (2017). It should be noted that the dry room examined here is oversized for a laboratory scale production. Only eight cells can be produced in one campaign (1.5 work days), regardless of the 100 m^2^ surface area of the dry room. In addition, there is a difference in the throughput considered in the studies (Thomitzek et al., 2019a; Yuan et al., 2017), which has a decisive impact on the energy demand per cell or Wh cell storage capacity. A sensitivity analysis is carried out in the following chapter to investigate the impact of the throughput. Moreover, the dew point temperature of the examined dry room at the BTC is −70°C, whereas Thomitzek et al. (2019a) indicate a constant dew point of −40°C and −60°C. However, the fact that the dry room has the highest energy requirement corresponds to the studies examined, insofar as the dry room was taken into account.

The relatively high share of the energy requirement for coating and drying compared with the other processes corresponds to the literature. However, in this study the energy requirement of 32 Wh is rather low, in particular when compared with Thomitzek et al. (2019a). Due to the lack of transparency of the studies in the literature review, it is hardly possible to identify a precise source of the difference here. However, the lowest energy demand, provided by Pettinger and Dong (2017), is mainly caused by the industrialized production facility. The impact of the production scale on the energy demand is discussed in more detail in the following chapter.

In the case of the process step formation, however, the energy requirement in this work is remarkably high compared with the other studies. The possible reasons for this, such as the cycler's own high energy requirement, are discussed in more detail in the following section.

The comparably high share in the category “others” in the case study is based on the consideration of the air conditioning of the formation room. It is not clear if this is also considered by the other studies and if so, where the energy demand is assigned.

## Discussion and sensitivity analysis

**Figure 6:**
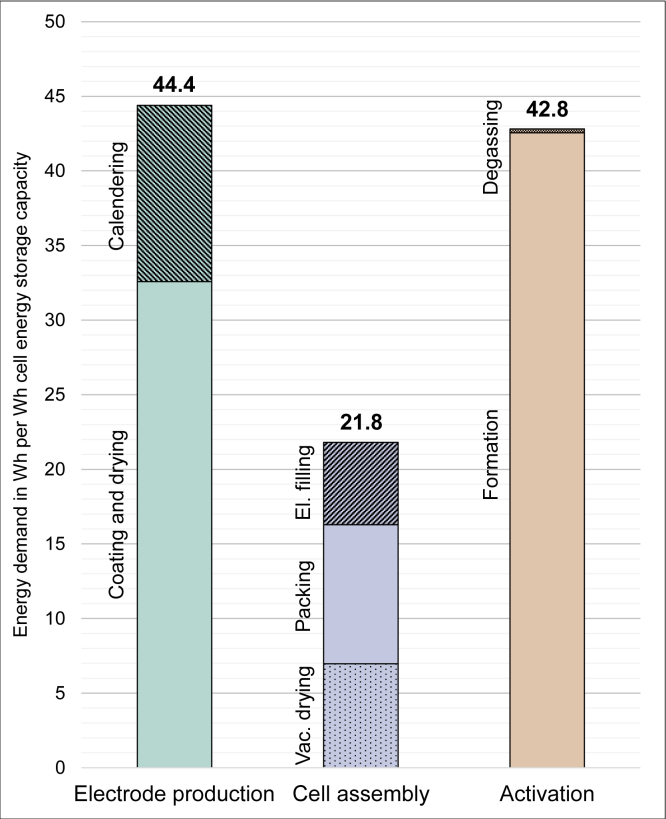
Energy demand in Wh per Wh cell energy storage capacity according to production category

Dividing the results into the superordinate categories of electrode production, cell assembly and activation, results in the diagram displayed in Figure 6. As coating and calendering are part of electrode production, the energy requirement is the highest, with 44.4 Wh per Wh cell energy storage capacity. Due to the formation, the energy requirement for activation is just slightly lower at 42.8 Wh per Wh cell energy storage capacity. Cell assembly with 21.8 Wh per Wh cell energy storage capacity requires only half the energy demand of electrode production. The low share allocated to cell assembly can be explained by the short process time (Thomitzek et al., 2019a). This might also be caused by the fact that there is no stacking considered, because this is performed manually at the BTC. As already mentioned, the three process steps of formation, coating, and calendering are responsible for 79.9% of the energy demand of cell production processes on laboratory scale and are discussed in more detail below.

### Formation

The high amount of required energy for the formation of the cell is based on the high degree of self-consumption through the cycler itself. The operating power of the cycler itself is 0.5 kW. This results from a formation time of 24 h, divided by the number of cells cycled simultaneously (four cells) in a demand of 39.4 Wh per Wh cell energy storage capacity equaling 3.00 kWh per cell. This represents 93% of the determined value for the total energy demand during formation. It is worth noting that no data could be found regarding this topic. A possible reason for the high energy demand of the cycler is based on its particular technical performance characteristic. Because the cells are produced for research purposes at laboratory level, a high precision and functionality of the cycler is required. This can lead to an increased energy demand. Furthermore, in industrial cell production more than four cells can be cycled at the same time, leading to a reduction in the energy requirement of the cycler per cell. In addition, there is a high variation in the formation process performed in the industry. Three full charging and discharging processes are performed in the analyzed laboratory production step. In contrast, several producers only perform until the SEI is formed (Korthauer, 2018). Another crucial point is that unlike the BTC, in large industrial plants the energy gained during discharge is returned to the grid, leading to energy recovery effects and thus to the reduction of energy demand. Nevertheless, according to Schönemann (2017), cell formation and aging are among the most energy- and time-intensive processes in cell production, which is in line with the findings of this study. According to Davidsson Kurland (2019), Northvolt expects to use up to 20% of the required energy of cell manufacturing only for cell formation.

### Coating

Coating requires a high energy input, which has been discussed in the literature repeatedly. It is even identified as the production step with the highest energy demand (Emilsson and Dahllöf, 2019; Pettinger and Dong, 2017; Thomitzek et al., 2019a, 2019b; Yuan et al., 2017). The reason for this is the high drying temperature of up to 130°C (Thomitzek et al., 2019a, 2019b) and the high evaporation enthalpy of the used solvents (Pettinger and Dong, 2017). In general, the energy requirement is higher for the cathode. Although the evaporation enthalpy of NMP (used in the cathode) is lower than that of water (often used in the anode), NMP has a higher boiling temperature (Pettinger and Dong, 2017). A very decisive reason for the increased energy consumption of electrodes with NMP instead of water is the air volume that has to be heated. Due to the explosiveness of NMP, a large excess of air has to be used to ensure that the maximum allowable NMP concentration in the air is not exceeded. The heating of this additional air volume leads to an increased energy requirement (Ahmed et al., 2016).

### Dry room and sensitivity analysis for scaling-up production volumes

**Figure 7:**
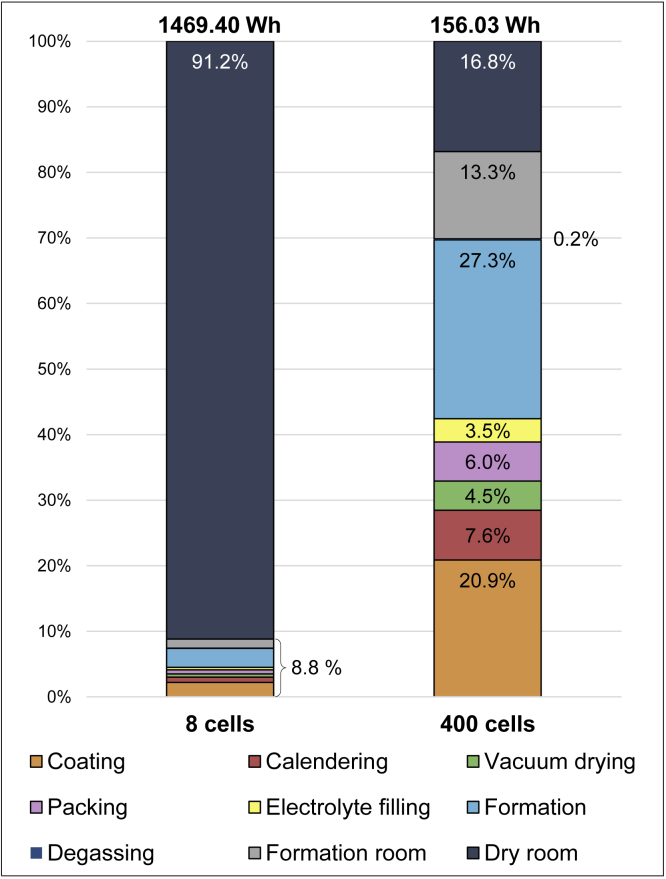
Comparison of energy demand in Wh per Wh cell energy storage capacity with different cell production throughputs

With 91.2%, the dry room accounts for the largest share of the required total energy for cell production and is, therefore, the most critical process in our investigation. Thomitzek et al. (2019a) explain this high share in their work with the semi-industrial character of the research establishment. This also comes true for this study, where the dry room has an even higher share. The comparable low throughput (eight cells in one operation equaling 12 h) due to the laboratory character and the long processing time consequently increases the energy demand per cell and therefore per Wh cell energy storage capacity. The dry room seems to be overdimensioned for research purposes. This significant impact of the throughput on the energy demand per cell is demonstrated clearly by the following sensitivity analysis. Yuan et al. (2017) have distributed the energy requirement for the dry room with a similar input power among 400 cells per day. In contrast, only eight cells are used for distribution in this assessment, as this equals the number of cells produced in one campaign. Assuming the same number of cells produced as Yuan et al. (2017), the demand per cell would decrease to 2 kWh per cell, which equals a demand of 26.27 Wh per Wh cell energy storage capacity. In comparison, Yuan et al. (2017) report a demand of 31.2 Wh. The changes in the total energy demand, including the shares of each production step for both throughput scenarios are displayed in Figure 7. The left bar illustrates the actual measured production with a maximum of eight cells. The right bar illustrates production with a throughput of 400 cells in the dry room. The total energy required for the dry room is therefore distributed over 400 cells in the right bar compared with eight in the left bar. As a result, the dry room accounts for only 16.8% instead of 91.2% of the total energy requirement in the left bar. Thus, by increasing the throughput of the cells by a factor of 50, the energy demand of the dry room per cell decreases by 81.58%. In this scenario, the total energy requirement for one cell would decrease to 156.03 Wh per Wh cell energy storage capacity.

**Figure 8:**
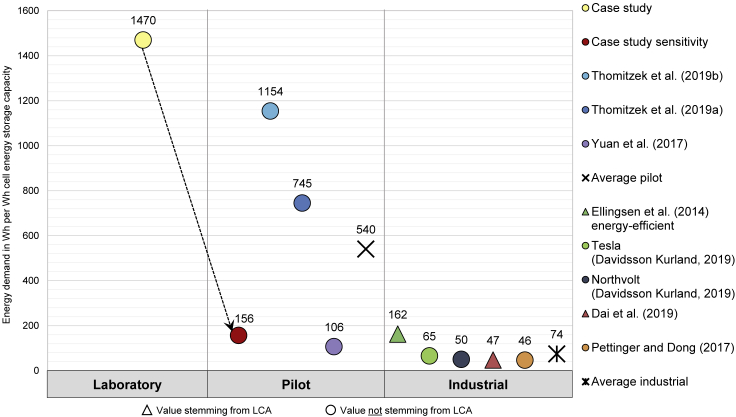
Impact of production volumes on the energy demand in Wh per Wh cell energy storage capacity for LIB cells according to selected studies

Overall, the energy demand obtained for a laboratory scale in this study is much higher when compared with the literature. This is caused by the lower cell manufacturing throughput as demonstrated by the exemplary analysis in Figure 7. A typical industrial cell production has an energy requirement several orders of magnitude lower in relation to a laboratory scale production (Li et al., 2014). The energy requirement per cell decreases significantly with increasing production volumes, according to the values stated in the literature and as shown in Figure 8, which is inspired by Davidsson Kurland (2019). This high dependence of the energy demand on the throughput, particularly for the dry room, was stated by Dunn et al. (2015) and Yuan et al. (2017). In addition, the dew point temperature of −70°C of the examined dry room is considerably lower compared with other studies, which have a dew point temperature of −40°C up to −60°C (Thomitzek et al., 2019a; Schönemann, 2017).

### Correlation between energy demand and production scale

The dependence of the energy demand on the throughput and thus on the production scale can be seen again in Figure 8, where the energy demand per cell energy storage capacity from different studies is shown. Values symbolized as triangles stem from LCAs, and values displayed as circles are determined independently from LCAs. Only the energy required to manufacture a single LIB cell is taken into account, thus excluding housing, BMS, or any similar component. In addition, the provided energy demands are categorized in laboratory, pilot, and industrial scale production. For a laboratory scale cell production, only the value of this study is available. For a pilot scale production the LCA-independent values from the studies by Thomitzek et al. (2019b), Thomitzek et al. (2019a) and Yuan et al. (2017) are pictured. Among the values reported for pilot scale production, there are major variations. Particularly the value of Thomitzek et al. (2019b) is striking, as it shows by far the highest value in that category. Although no information is given on the production volume, it is assumed that a low throughput is responsible for this value due to the research character of the facility. However, Thomitzek et al. (2019a) report a production volume of 1,450 cells per year (consisting of 280 days), which could be explained by the research character of the production and a probably lower production volume than Yuan et al. (2017). However, the exact production volume of the pilot scale facility of Yuan et al. (2017) remains unclear. Nevertheless, it is stated that the investigated dry room has a capability of 400 cells per day, which would lead to a production capability of 112,000 cells per year by assuming 280 days. The value obtained from the sensitivity analysis (case study sensitivity) of the previous section is also used in Figure 8 and labeled as pilot scale. Therefore, the red circle also symbolizes an energy requirement per Wh for a production with 400 cells per day.

For an industrial scale battery cell production, the LCA-independent values for Northvolt and Tesla provided by Davidsson Kurland (2019) and the energy demand reported by Pettinger and Dong (2017) are given. Industrial scale values stemming from LCAs are represented by the studies of Ellingsen et al. (2014) and Dai et al. (2019). In comparison to the values listed in the pilot scale, these energy requirements are considerably more homogeneous. Yet, the value provided by Ellingsen et al. (2014) is roughly three times higher than the other values reported in that production scale and even higher than the pilot scale value provided by Yuan et al. (2017) and thus reveals the highest difference. However, this value was published in 2014, and more efficient production processes have since been developed. Ellingsen et al. (2014) mention that the technology is at an early stage of development and that future production volumes are expected to be larger and therefore more energy efficient, yet without revealing the exact production numbers. Pettinger and Dong (2017) state a production capability of 1.5 million cells per year (consisting of 280 days) for their facility. Davidsson Kurland (2019) indicates 328 GWh cell manufacturing capacity for Northvolt and 35 GWh cell manufacturing capacity for Tesla. In the study of Dai et al. (2019) a production capacity of 40,000 cells per day is given equaling an amount of 2 GWh cell manufacturing capacity per year.

Overall, the highest value refers to the laboratory scale production determined within this work. The lowest value is related to the industrial scale production of the study by Pettinger and Dong (2017). With a difference of 1424 Wh, the energy requirement on a laboratory scale corresponds to the 32-fold of an industrial scale production. The average value for a pilot scale production calculated with the values from Figure 8 is 540 Wh and for an industrial scale production 74 Wh.

As indicated above and shown in Figure 8, the energy requirement per Wh in industrial scale manufacturing is several orders of magnitude lower than that of laboratory scale manufacturing. The comparison of the studies shown in Figure 8 reveals clearly the strong relation of the energy demand per Wh to the production throughput. A larger production volume entails economies of scale. For example, the energy required for the heating of machinery or for the spatial environment such as the dry room can be distributed among more cells and thus reduce the demand per cell or Wh storage capacity.

Furthermore, in industrial processes, there are additional synergy effects that have an influence on the energy requirement. This correlation was investigated in detail by, e.g., Shibasaki et al. (2006). Processes in pilot and especially in laboratory scale are often isolated and independent from each other, in contrast to industrial processes, which in battery manufacturing are highly automated coherent production lines. This interconnection of the process steps reveals synergy effects, for example, waste heat from one process can serve as an input to a thermal energy source within the same production chain. A more concrete example of this has already been given in the previous section “Formation,” because in industrial productions the energy when discharging the cell is returned to the system. Hence, interconnected industrial scale processes reveal a greater opportunity for energy recovery effects and thus a lower energy requirement than is the case for laboratory or pilot scale processes (Piccinno et al., 2016; Shibasaki et al., 2006).

In addition, industrial scale productions are far more efficient than those on a laboratory scale. The utilization rate of the respective machines is optimized and capacities are thereby fully utilized. As a result, energy requirements for switching on and off machines (e.g. heating up) or energy consumption in standby mode can be prevented (Shibasaki et al., 2006). This efficiency of industrial manufacturing is also evident in the low scrap rate. For example, Pettinger and Dong (2017) report a scrap rate of 8% for the production line investigated in their study. Although it is not possible to make a quantified statement on the scrap rate for the laboratory scale production on which this work is based, it can be said that it is considerably higher. One reason for these differences lies in the divergent goals of the various production types. A production line on an industrial scale aims for cost efficiency, resulting in optimized processes in terms of time and energy use. Thus, the production line is adjusted and optimized for a specific production case, and the parameters are fixed. However, laboratory level processes are used for scientific research in order to obtain fundamental knowledge of different materials, machineries, or processing techniques. Therefore, the smaller laboratory scale plants are needed to make larger scale production feasible in the first place. Hence, the materials used, cell sizes, or processing speeds change frequently, and there are no constant parameters on which the process has been optimized (Piccinno et al., 2016; Shibasaki et al., 2006). This can lead to more failures in laboratory scale processes, as was the case in this study with coating and drying (see 3.1 in supplemental information). Here, the aluminum foil ripped several times during the process, so that the foil had to be re-clamped multiple times. As a result, part of the foil but also of the slurry and consumed energy were wasted.

### Transferability of results to new battery technologies in an early development stage

The results of this work can be applied in particular to SIBs. They are often seen as a complementary technology to LIBs (Vaalma et al., 2018) and show similarities in functionality (Sawicki and Shaw, 2015). Peters et al. (2016) assumed (based on expert judgment) in their LCA on SIBs that the results of LIB production can be transferred to SIB. As an example, the production of SIBs also requires a dry room. Therefore, this work can serve as a basis for determining the amount of energy needed to produce an SIB on a laboratory scale.

### Conclusion and outlook

The aim of this work was to conduct a bottom-up analysis of the energy demand of an LIB production on a laboratory scale and to contrast the results with recent literature considering different levels of cell production (laboratory, pilot, and industrial scale). Thus, this work provides both lab scale primary data and also a range of the energy demand for LIB production available in the literature from lab through pilot to industrial scale. Specifically, the laboratory scale LIB cell production at the KIT is used as a reference. Multiple measurements were carried out on the relevant machines and facilities of the cell production line. The results of this work show general similarity with the values obtained from the literature and provide an in-depth view of the differences between laboratory, pilot, and industrial scale production. These results are examined in detail and some significant differences can be identified. Overall, the findings can be summarized as follows:

• The highest energy demand in an LIB cell production is caused by the dry room, due to the high power needed for dehumidifying and cooling the air. However, the demand in this work is much higher than in literature, mainly due to the low throughput and overdimensioning of the dry room on a laboratory scale.
• On the process side, the highest energy demand is caused by formation, coating, and calendering.
• The high energy requirement for the formation of the cells can be explained in this work by the high energy requirement for operating the cycler and the missing energy recovery.
• Coating and drying also contribute significantly to the energy demand, due to the high drying temperatures.
• Overall, a higher demand than in literature can be found within this work. This is mainly due to the laboratory character of the production and the resulting low throughput. Due to the lower throughput, the machines' own energy requirements cannot be redistributed to more cells, causing a higher energy demand per cell. Synergy effects of industrial scale production facilities that reduce the energy demand, are missing.
• The energy-intensive steps underlying this laboratory scale production are identical to those of other production scales. Accordingly, it is possible to identify and analyze these key processes at this production level.

In summary, this work highlights the importance of studies on the sustainability of LIBs, while addressing the lack of reliable data on the energy demand for the production of an LIB cell on different levels (laboratory, pilot, and industrial scale). The values available in the literature differ greatly, and the framework conditions within those studies are not fully available, especially in earlier studies. This work shows that significant differences on the process level exist among the studies. The reason for these specific differences remains unknown and thus requires further research. Consequently, a comparison of the results and a valid statement on the energy demand of an LIB cell production is only possible to a limited extend.

Comprehensive and transparent data are a crucial precondition to carry out robust studies on LIBs, particularly when conducting LCAs, and there is a high demand for such primary data. This paper provides both initial values for further assessments, based on a condensed overview of relevant studies. Most importantly, the consideration of the energy requirements in relation to the respective production volume or production level (laboratory, pilot, or industrial) represents the distinctive approach of this study. It was shown that a detached consideration of the energy demand from the production level would not be appropriate, as the energy demand per Wh decreases with increasing production numbers. This is the only existing study filling the gap for a laboratory scale energy demand analysis for LIBs by providing in-house measurements in a transparent manner. The provided primary data can serve as a starting point for early stage sustainability assessments for new cell types under investigation.

The availability of a detailed description of the measurement performance, as well as the high transparency in the processing of the measurement results, distinguishes this work from many of the other examined studies. Overall, the results can hardly be generalized and have to be related to the given conditions but should provide a starting point for further related research. In particular, this includes the scalability of the laboratory scale results to other production scales, such as the pilot or industrial level. The obtained findings can not only serve as an initial guide for scaling-up laboratory production and examining scaling effects but also be applied to an LCA of a new battery technology with a low TRL in laboratory scale.

Further research will be carried out, particularly with regard to the influence of various production parameters on the energy requirement. However, for future analyses, measurements on a coherent production chain, but also transparent and accessible data in the literature, would be desirable.

### Limitations of the study

The measurements are based on laboratory scale production processes of the KIT20 pouch cell. Deviations may occur when the measurements are repeated. As the energy demand of the dry room was calculated based on values for May 2019, the energy demand could differ from that of another month or season.

### Resource availability

#### Lead contact

Further information and requests for resources and materials should be directed to and will be fulfilled by the lead contact, Merve Erakca (merve.erakca2@kit.edu).

#### Materials availability

This study did not generate new unique reagents.

#### Data and code availability

The published article includes all datasets generated or analyzed during this study.

## Methods

All methods can be found in the accompanying transparent methods supplemental file.

## Supplemental information

Document S1. Transparent methods, Figures S1–S6, and Tables S1–S9

## Footnotes

Supplemental information can be found online at https://doi.org/10.1016/j.isci.2021.102437.
