# Supplementary material for: Energy flow analysis of laboratory scale lithium-ion battery cell production
Source: iScience. 2021 Apr 16;24(5):102437. doi: 10.1016/j.isci.2021.102437 (PMC8102913; doi:10.1016/j.isci.2021.102437)
Supplement: Document S1. Transparent methods, Figures S1–S6, and Tables S1–S9 [file mmc1.pdf]

iScience, Volume *24*

Supplemental information

Energy flow analysis of laboratory scale lithium-ion battery cell production

Merve Erakca, Manuel Baumann, Werner Bauer, Lea de Biasi, Janna Hofmann, Benjamin Bold, and Marcel Weil

Transparent Methods

1 KIT 20 Pouch Cell

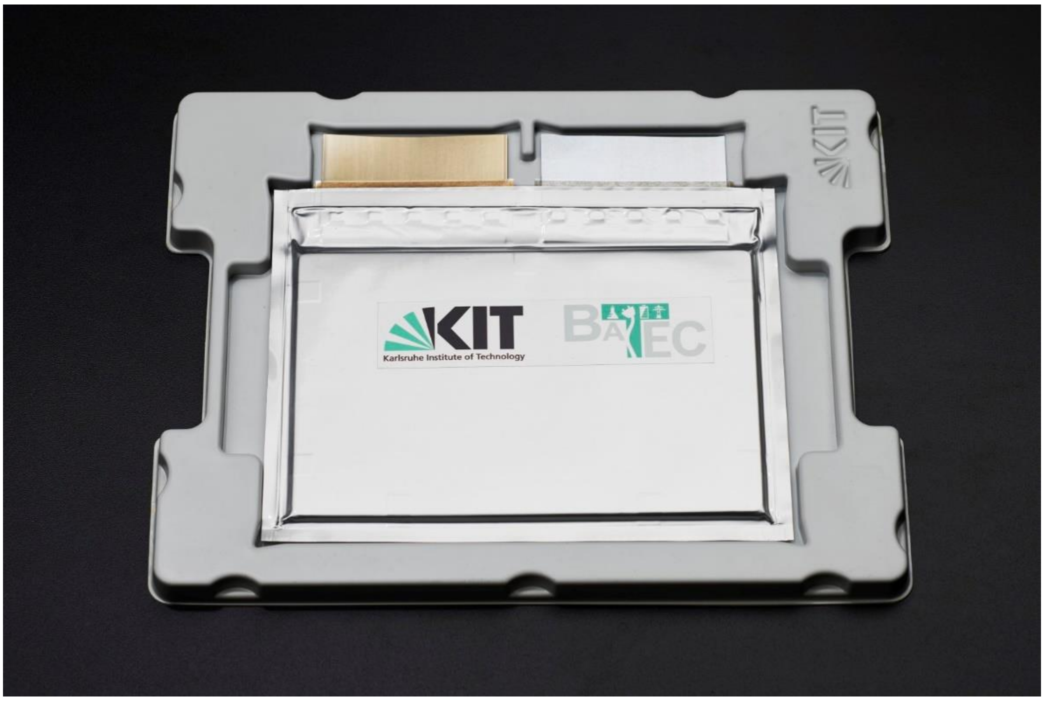

Figure S1 KIT 20 Pouch Cel, Related to System Boundaries and Assumptions (KIT 20 Li-ion cell, 2020)

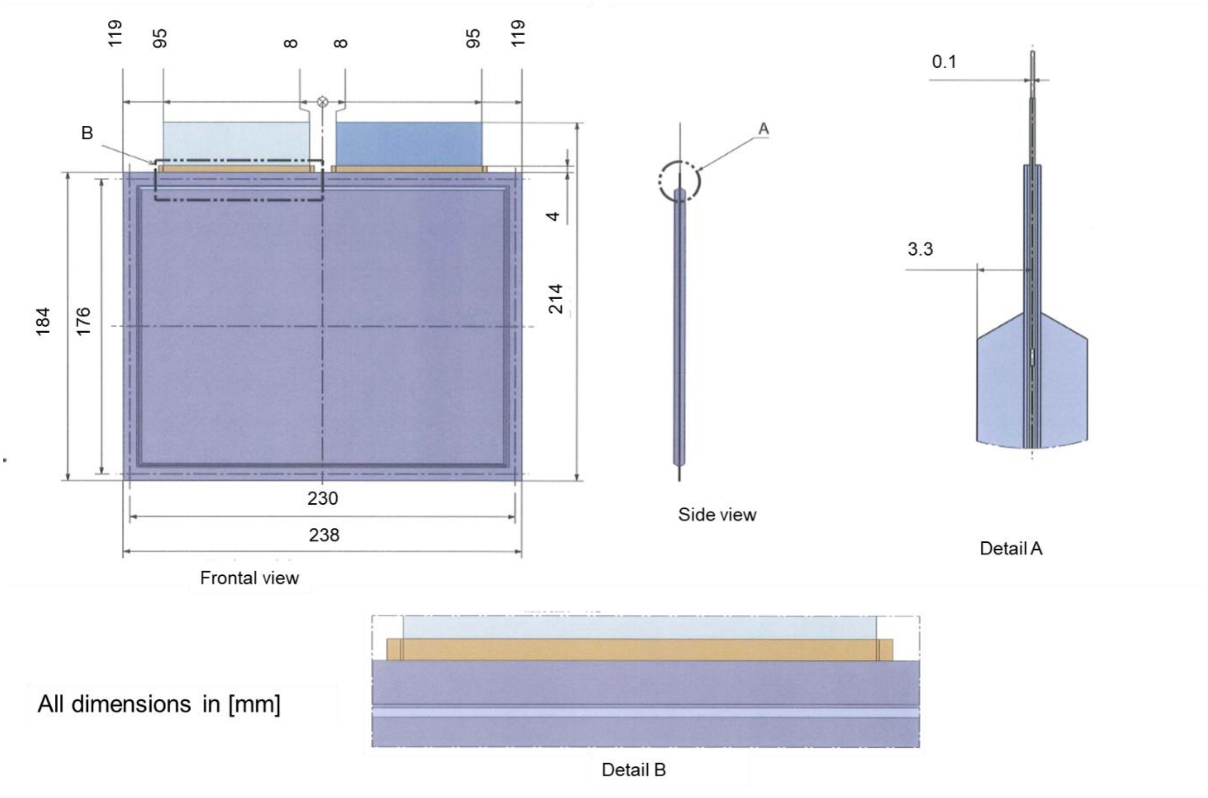

Figure S2 Dimensions of the KIT 20 Pouch Cell, Related to System Boundaries and Assumptions (KIT 20 Li-ion cell, 2020)

2 Measuring Device

The measuring device used in this work, pictured in figure S3, is called Tinytag Energy Logger by the company Gemini Data Logger. The device is able to record the voltage, power and current in three-phase operation by using Rogowski measuring coils. (Gemini Data Loggers (UK) Ltd., 2014) Based on Faraday's and Ampere's law, the alternating and high speed impulse currents are measured (Samimi et al., 2015).

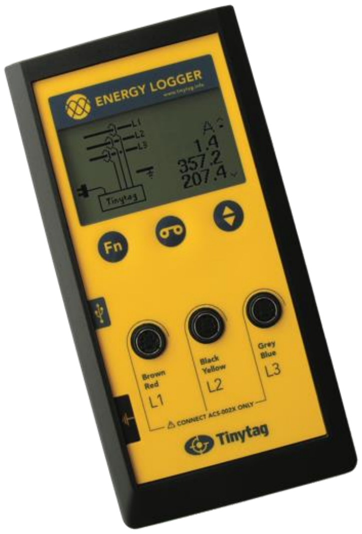

Figure S3 Measuring Device Tinytag Energy Logger, Related to Methodology (Gemini Data Loggers (UK) Ltd., 2014)

The flexible coils are wound around the power wires and connected to the Tinytag Energy Logger. The data logger scans current signals at a frequency of 5 kHz over a period of 100 ms and calculates the effective current flow and the actual power. The data logger can measure the current flow simultaneously on three wires, but the voltage can only be measured directly on one wire. The data logger generates equivalent voltage signals for the other two phases by delaying the measured voltage signal by one third and two-thirds of the main frequency.

Thus, the following constellation is assumed:

- The phases are offset by 120°.
- The voltage at all three phases is the same.
- Any harmonic distortions occur identically in all three phases.

With this constellation, an error probability of up to 1 % is possible. The logging interval can be adjusted from 30 s up to every ten days. (Gemini Data Loggers (UK) Ltd., 2014)

Within this work a logging interval of 30 s is selected for all measurements. The average of the logged power values for each process step during the relevant measuring time is calculated and multiplied with the relevant process duration to determine related energy demands.

In the case of two process steps, the energy logger could not be used since the corresponding machines are not connected to three-phase current, but connected to a socket. Therefore, an energy cost meter is used for determining the energy demand. For measuring, the device is interconnected between the socket and the machine. A display on the device shows the cumulative energy used, the cumulative recording time and the corresponding energy cost. For this device, it is not possible to set a logging interval or to display and record individual measurement values such as the operating power. Therefore, the energy demand is determined by the recorded value and scaled linearly for the relevant process duration.

3 Measuring Procedure

In the following, the process procedures and calculations, as well as the measurement trials and the related parameters and assumptions, are explained in detail.

3.1 Coating and Drying

For the measurements for coating and drying a cathode is coated with the parameters shown in figure S4.

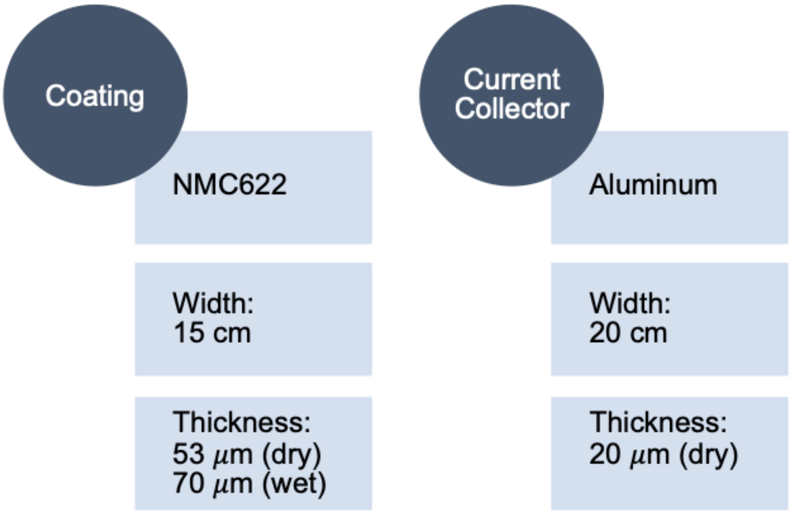

Figure S4 Parameters for Coating of the Cathode, Related to System Boundaries and Assumptions

After nine minutes of heating time, the desired temperatures for the two chambers (80°C and 120°C) are reached. The average operation power is 13.09 kW. Multiplying this value with the heating time of nine minutes results is an energy demand of 1.97 kWh for heating up the coating machine.

However, process-related difficulties occurred during this measurement. Specifically, these difficulties involve the tearing of the electrode carrier foil. Hence, the process is stopped several times and the aluminum foil is re-clamped. Higher coating speeds lead to an uneven distribution of the coating in our experiment. Therefore, the initial coating speed of 0.5 $m \cdot min^{-1}$ is successively reduced to 0.3 $m \cdot min^{-1}$ as shown in table S1, leading in 4 trials each with a lower coating speed and therefore a higher energy demand per meter.

Table S1 Energy Demand for Coating, Related to System Boundaries and Assumptions

Parameter	Unit	Trial 1	Trial 2	Trial 3	Trial 4
Speed	$m \cdot min^{-1}$	0.50	0.40	0.35	0.30
Time	min	9.0	16.5	30.0	21.0
Length	m	4.5	6.6	10.5	6.3
Operating power	kW	6.26	6.37	6.17	6.32
Demand without heating	kWh	0.94	1.75	3.09	2.21
Demand with heating	kWh	1.00	1.84	3.22	2.30
**Energy demand per meter**	**$kWh \cdot m^{-1}$**	**0.22**	**0.28**	**0.31**	**0.36**

The coating length is calculated based on the coating speed and time. The operating power for each trial is the mean value from the measured power values during the coating time. By multiplying the operating power with the coating time, the energy demand without heating is obtained for each trial, shown in the third row in table S1.

Nevertheless, the above determined total energy demand of 1.97 kWh for heating has to be redistributed for which the assumptions shown in table S2 are made.

Table S2 Assumptions for Redistributing Heating Energy, Related to System Boundaries and Assumptions

Parameter	Value
Beginning of coating	10:40
End of coating	17:24
Theoretical coating time	404 min
Average coating speed	0.36 m·min^{-1}
Coating length	147.3 m

The total coating duration equals 404 min, which corresponds to the total time required for the trials. Based on the different coating speeds, an arithmetic mean of 0.36 m·min^{-1} is calculated for the coating speed. By multiplying the presumed coating time and the calculated coating speed, a theoretical coating distance of 147.3 m is obtained.

By dividing the total energy requirement of 1.97 kWh for heating with the theoretical coating distance of 147.3 m, an energy demand of 0.01 kWh·m^{-1} for heating is determined. This value is redistributed depending on the trials and the corresponding coating length and merged with the previously determined energy demand without heating, shown in the penultimate row of table S1. By dividing this energy demand with the corresponding coating length, the energy demand per meter for each trial is determined. Therefore, the resulting mean energy demand per meter for cathode coating is 0.30 kWh·m^{-1}.

Since measurements are carried out on cathodes, this value distinguishes from the energy demand for coating anodes. As mentioned in chapter System Boundaries and Assumptions the energy demand for anodes is approximately 15 % less than for cathodes (Pettinger, 2019), resulting in an energy demand of 0.25 kWh·m^{-1}.

Those energy demands (0.30 kWh·m^{-1} for cathode and 0.25 kWh·m^{-1} for anode) are multiplied by the length of one electrode sheet and then multiplied by the amounts of sheets for one cell. As a result energy demands of 1.31 kWh for the cathodes and 1.17 kWh for the anodes are gained leading to a total energy demand of 2.48 kWh per cell for coating and drying. Based on the cell weight of 540 g and a gravimetric energy density of 141 Wh·kg^{-1} an energy demand of 32.57 Wh per Wh cell storage capacity is obtained.

3.2 Calendering

For the measurements during this process, cut parts of cathodes with the cathode material NMC111 are used rather than continuous electrodes. In total, 15 strips with a length of approximately 30 cm each are manually supplied to the calender. Before calendering, the calender is heated up to 50°C, requiring 105 minutes. The average operating power is 2.05 kW leading to an energy demand of 3.58 kWh to heat up the calender.

In order to determine the energy requirement of the calender six trials are carried out. Three trials are carried out with a calendering speed of 1 m·min^{-1} and the other three with a calendering speed of 5 m·min^{-1}. Each trial is conducted until all 15 strips are calendered. The results of the individual trials are listed in table S3.

Table S3 Energy Demand for Calendering, Related to System Boundaries and Assumptions

Parameter	Unit	Trial 1	Trial 2	Trial 3	Trial 4	Trial 5	Trial 6
Speed	m·min^{-1}	1.0	1.0	1.0	5.0	5.0	5.0
Time	min	7.5	4.5	5.0	1.0	1.0	1.8
Length	m	7.5	4.5	5.0	5.0	5.0	8.8
Operating power	kW	1.03	1.01	1.05	1.12	1.00	1.06
Demand without heating	kWh	0.13	0.08	0.09	0.02	0.02	0.03
Demand with heating	kWh	0.76	0.46	0.51	0.44	0.44	0.77
**Energy demand per meter**	**kWh·m^{-1}**	**0.10**	**0.10**	**0.10**	**0.09**	**0.09**	**0.09**

The duration of the measurement differs due to the manual supply. However, the operating power is highly consistent in all trials and averages 1.05 kW. The measurements indicate no difference in power, whether operating empty or actually calendering a strip. Similarly, the calendering speed hardly affects the operating power. The calendered length is calculated using the calendering speed and the time, assuming that this equals the calendered length when using a continuous electrode coil.

The energy required for heating is redistributed among the trials according to the calendered length. At a speed of 1 m·min^{-1} the total energy requirement is 0.10 kWh·m^{-1} and at 5 m·min^{-1} the requirement is 0.09 kWh·m^{-1}. In the further course 0.10 kWh·m^{-1} is used as the energy requirement for calendering on a laboratory scale.

Again this energy demand is multiplied by the length of one electrode sheet and then multiplied by the amounts of sheets for one cell. As no difference in the energy demand for calendaring cathodes and anodes is assumed a total energy demand of 0.9 kWh per cell is gained. Based on the cell weight of 540 g and a gravimetric energy density of 141 Wh·kg^{-1} an energy demand of 11.82 Wh per Wh cell storage capacity is obtained.

3.3 Vacuum Drying

Before storing the cells in the vacuum dryer, the pre-cut electrode and seperator sheets are manually stacked and fixed. A cost meter is used for measuring the energy requirement of vacuum drying (since the device is directly connected to a power socket). Two measurements are carried out. The first measurement is performed for 19.4 hours on an empty vacuum dryer, the second for 21.1 hours on a vacuum dryer containing pouch cells.

For further analyses, the average of both values, 6.36 kWh, is used. This value is divided by 12, which equals the usual number of cells dried simultaneously in the vacuum dryer, resulting in an energy demand of 0.53 kWh per cell, which equals 6.96 Wh per Wh cell storage capacity.

3.4 Packing

In order to provide a better overview, this process is subdivided into the steps connecting current collector, sealing of the pouch foil and final sealing of the pouch foil after degassing.

Connecting Current Collector

The two contact tabs (aluminum for cathodes, nickel for anodes) have to be cut off before connecting by ultrasonic welding, as their length has to be fitted to the corresponding cell. Each collector is equipped with five welding spots. Only the welding process itself proceeded automatically. Thus, the cells are moved manually after each welding spot. The entire welding process lasts eight minutes for cell 1 and nine minutes for cell 2 as displayed in table S4.

Table S4 Energy Demand for Contacting Current Collector, Related to System Boundaries and Assumptions

Parameter	Unit	Cell 1	Cell 2
Time	h	0.13	0.15
Operating power	kW	0.15	0.14
**Energy demand**	**kWh**	**0.02**	**0.02**

The mean operating power for cell 1 is 0.15 kW and for cell 2 0.14 kW resulting in an energy demand of 0.02 kWh for cell 1 and for cell 2.

Sealing Pouch Foil

Before the pouch cell can be sealed, the sealing machine has to be heated for 15 minutes. The average operating power for heating is 2.34 kW. Multiplying this value with 15 minutes of heating time yields an energy requirement of 0.58 kWh. Since two cells are produced, this corresponds to an energy demand of 0.29 kWh per cell for heating. After reaching the desired sealing temperature, a sample sealing is performed to ensure an accurate quality of the sealed seam. The sample sealing comprises the sealing of two sides of a pouch foil. Given a duration of 1.5 minutes and an output of 0.67 kW, this results in an energy requirement of 0.02 kWh in total for sample sealing, equaling 0.01 kWh per cell. After this, the actual sealing of the pouch foil of the two cells starts. Table S5 displays the sealing of the three sides of the pouch foil in more detail.

Table S5 Energy Demand for Sealing Pouch Foil, Related to System Boundaries and Assumptions

Parameter	Unit	Cell 1	Cell 2
Time	h	0.08	0.09
Operating power	kW	0.92	0.87
**Energy demand**	**kWh**	**0.07**	**0.08**

The sealing time for cell 1 is 4.5 minutes, and 5.5 minutes for cell 2. Using the operating power for both cells, 0.92 kW for cell 1 and 0.87 kW for cell 2, yields in an average energy demand of 0.07 kWh per cell. Based on this, a total energy requirement of 0.37 kWh per cell for the sealing of the pouch foil is calculated.

Final Sealing Pouch Foil

After degassing, the remaining side of the pouch cell has to be sealed and the gas pocket has to be cut off manually. This requires a renewed heating of the sealing machine. Thus, an energy demand of 0.29 kWh per cell for heating is again necessary. Since the previously determined energy demand of 0.07 kWh per cell includes the sealing of three sides, this value is divided by three to obtain the energy demand for sealing one side, resulting in 0.025 kWh per cell. Accordingly, the required energy demand for the final sealing process of the pouch cell is 0.32 kWh per cell.

By adding the results of all three processes, a total energy demand of 0.71 kWh per cell is obtained for packing. This equals an energy demand of 9.32 Wh per Wh cell energy storage capacity.

3.5 Electrolyte Filling

As electrolyte filling is a comprehensive manufacturing process, it is divided into subprocesses flushing procedure 1, flushing procedure 2, flushing procedure 3, electrolyte filling, flushing procedure 4 and flushing procedure 5.

Flushing Procedure 1: Isopropanol

During the first flushing procedure, the tubes are cleaned with isopropanol for two minutes. The mean operating power is 1.74 kW leading to an energy demand of 0.05 kWh.

Flushing Procedure 2: Propylene Carbonate

With the second flushing process, the tubes are cleaned with propylene carbonate for two minutes. The average operating power is 1.19 kW resulting in an energy demand of 0.04 kWh for this process.

Flushing Procedure 3: Electrolyte

The final flushing procedure is carried out with the electrolyte itself in order to remove any residues of the other materials and to avoid them entering the cell. The process is carried out again for two minutes, with a mean operating power of 1.20 kW resulting in an energy demand of 0.04 kWh.

Electrolyte Filling

After cleaning the tubes, the electrolyte filling starts. The procedure is carried out for five minutes with a mean operating power of 1.09 kW for cell 1 and 1.10 kW for cell 2, resulting in an energy demand of 0.09 kWh per cell as displayed in table S6.

Table S6 Energy Demand for Electrolyte Filling, Related to System Boundaries and Assumptions

Parameter	Unit	Cell 1	Cell 2
Time	h	0.08	0.08
Operating power	kW	1.09	1.10
**Energy demand**	**kWh**	**0.09**	**0.09**

Flushing Procedure 4: Isopropanol

After filling the electrolyte, the tubes must be cleaned again with isopropanol. Again, a flushing procedure is carried out for two minutes. The mean operating power of 1.16 kW causes an energy demand of 0.04 kWh.

Flushing Procedure 5: Propylene Carbonate

The final step of the process of electrolyte filling is the final flushing with propylene carbonate for two minutes. The energy demand is 0.05 kWh as a result of the average operating power of 1.53 kW.

The indicated values for all steps are summarized in table S7.

Table S7 Energy Demand for Flushing Procedures, Related to System Boundaries and Assumptions

Parameter	Unit	Flushing 1	Flushing 2	Flushing 3	Flushing 4	Flushing 5
Time	h	0.03	0.03	0.03	0.03	0.03
Operating power	kW	1.47	1.19	1.20	1.16	1.53
**Energy demand**	**kWh**	**0.05**	**0.04**	**0.04**	**0.04**	**0.05**

Warming Cabinet

After filling the electrolyte, the cells are stored at elevated temperature for 24 hours in a climate chamber located in the air-conditioned formation room. This ensures that the electrolyte is distributed homogeneously in all layers of the cell stack prior to formation. The energy requirement for storing the cells in the warming cabinet is measured by the energy cost meter for 3.88 hours. The measured value of 0.32 kWh is then scaled up to the real process duration of 24 hours, resulting in a demand of 1.97 kWh. This value is divided by nine, as this represents the maximum number of cells that can be stored, resulting in a demand of 0.22 kWh per cell.

A total energy demand of 0.42 kWh per cell is obtained for electrolyte filling. For this value the energy demand of the warming cabinet is added to the previously determined energy demand for the flushing procedures and the electrolyte filling. This value is then dividing according to the number of cells produced (in this case two). This corresponds to a value of 5.52 Wh per Wh cell energy storage capacity.

3.6 Formation

The cell cycling consists of three charging and discharging processes in the air-conditioned formation room with a duration of 24 hours. The energy demand for the charging and discharging process is determined by adding the individual energy demand of each cycling process, resulting in an energy requirement of 0.24 kWh for cycling as displayed in table S8.

Table S8 Energy Demand for Formation, Related to System Boundaries and Assumptions

Parameter	Unit	Value
Time	h	24
Energy demand cycling	kWh	0.24
Energy demand device	kWh	3.00
**Total energy demand**	**kWh**	**3.24**

The energy demand for each cycling process is calculated by the BTC. The cycling device has an operating power of 0.50 kW and an operating time of 24 hours, resutling in a demand of 12 kWh . This demand is divided by the number of cells simultaneously attached to the cycler. As four cells are cycled, this corresponds to a demand of 3.00 kWh per cell leading to a total energy requirement of 3.24 kWh per cell. This value equals an energy demand of 42.55 Wh per Wh cell energy storage capacity. Remarkably, the majority of the energy demand is not due to the execution of the cycling process, but to the cycling device itself (due to e.g., electronics for a detailed measuremnet of the cell performance).

3.7 Degassing

Degassing marks the final process step of the studied LIC production on a laboratory scale and is carried out on the same machine as electrolyte filling. By operating for 1.5 minutes with an operating power of 0.96 kW an energy demand of 0.02 kWh per cell or 0.26 Wh per Wh cell storage capacity is obtained. After degassing the remaining gas pocket is removed manually by a slicer.

Table S9 Energy Demand for Degassing, Related to System Boundaries and Assumptions

Parameter	Unit	Cell 1	Cell 2
Time	h	0.03	0.03
Operating power	kW	0.96	0.96
**Energy demand**	**kWh**	**0.02**	**0.02**

3.8 Formation Room

The formation room requires a steady temperature of 25°C, hence the energy requirement of the air-conditioning system is measured. The measurement is carried out for 19.33 hours. As displayed in figure S5 there is little fluctuation in the operating power of the air-conditioning system.

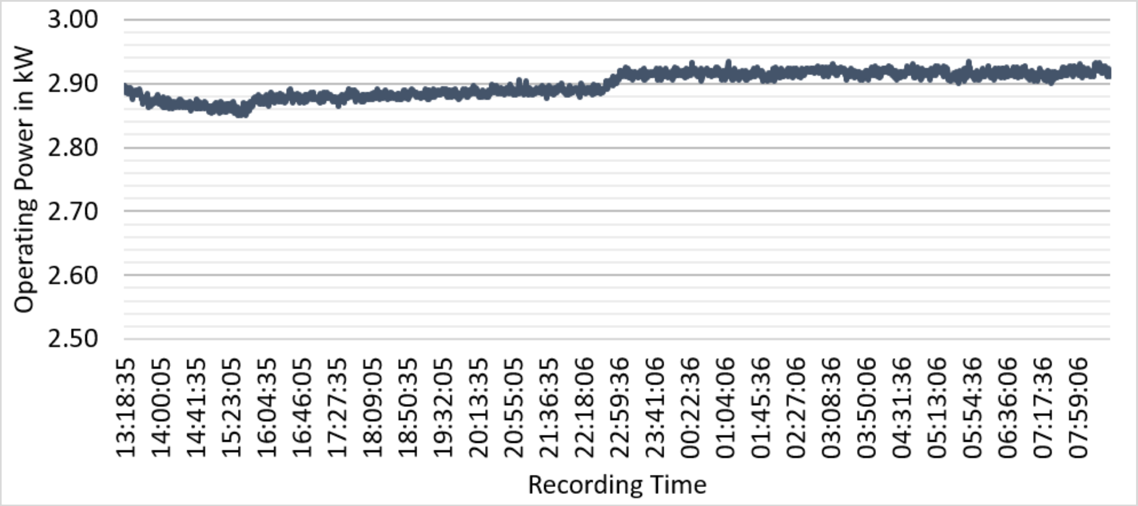

Figure S5 Operating Power of Air-Conditioner, Related to System Boundaries and Assumptions

Therefore, the mean operating power of 2.90 kW is determined and multiplied with 48 hours of formation time, resulting in an energy requirement of 139 kWh. The determined energy demand is divided by 88, which represent the maximum number of cells that can be cycled simultaneously in the formation room. Finally, an energy demand of 1.58 kWh per cell is calculated corresponding to a vlaue of 20.75 Wh per Wh cell energy storage capacity.

3.9 Dry Room

The energy requirement for the dry room is not determined by measurements, but by calculation. The load values for the entire BTC building are used for this purpose. The month of May 2019 is selected, as a lot of work has been carried out in the dry room during this period. First, the mean power value is calculated based on three days with switched-off dry room. This value is subtracted from the average power value of three days with a switched-on dry room. Thus, the difference represents the operating power of the dry room. The values for the switched-off dry room refer specifically to the days 05/01/19, 05/09/19 and 05/25/19. The values for the days with switched-on dry room refer to 05/16/19, 05/23/19 and 05/28/19. Figure S6 displays the difference in the load values of a day with switched-on dry room (orange) and a day with switched-off dry room (blue). The left diagram represents the days 05/25/19 (switched-off dry room) and 05/16/19 (switched-on dry room). Furthermore, the right diagram represents the days 05/09/19 (switched-off dry room) and 05/23/19 (switched-on dry room). As can be seen from the two diagrams, the differences in each are almost the same, confirming the assumption that the difference relates to the operating power of the dry room.

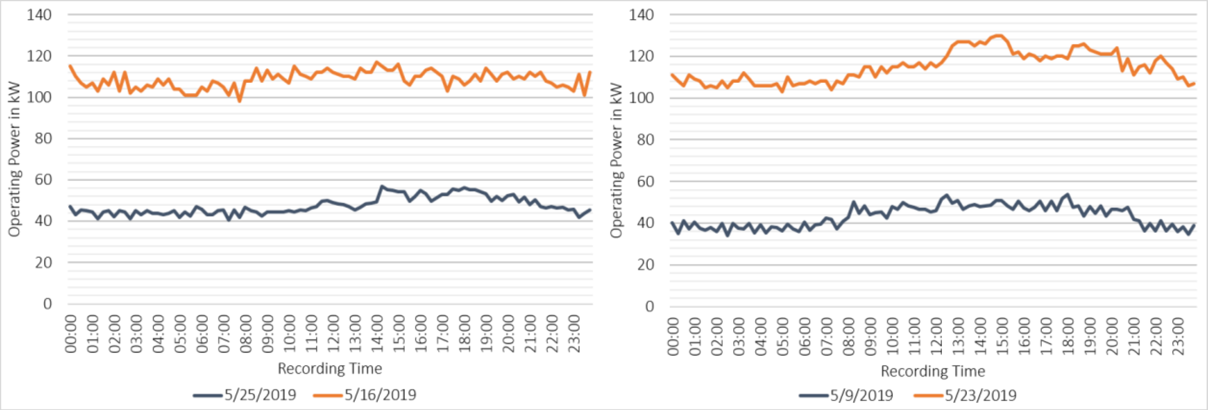

Figure S6: Operating Power of Days with Switched-on and Switched-off Dry Room, Related to System Boundaries and Assumptions

The average value for the load values of three days with switched-off dry room is 45 kW and for the days with switched-on dry room 112 kW, resulting in a difference of 68 kW. Yuan et al. (2017) state a power demand of 64.8 kW for the studied pilot scale dry room, validating the assumption made in this work. Based on an operating time of 12 hours, this results in an energy requirement of 814 kWh. Since eight cells can be produced within this period, an energy demand of 102 kWh per cell is obtained. This corresponds to the converted value of 1339.62 Wh per Wh cell storage capacity.

However, a number of additional parameters have a significant impact on the energy demand. One of the most important criteria for the power consumption is the amount of people in the dry room, as each person releases 100 to 120 $g \cdot h^{-1}$ of moisture into the environment. Furthermore, the seasons should be considered. Since the ambient air is more humid in summer, more energy is needed for dehumidification. Whereas in winter the ambient air is dry and less energy is needed for dehumidification. Fluctuations in the ambient air can also be caused by geographical differences. An additional influencing factor is the exhaust air. Due to the exhaust air from the machines and the arising exhaust gases, the air has to be constantly replaced with fresh air, which has to be dehumified and purified first (Eberhardt, 2019). Thus, the values determined by the measurements represent a snapshot of the data determined for the month of May and may differ from the annual average value for energy consumption.

Supplemental References:

Eberhardt, K., 2019. Telephone Interview: Energy Demand of a Dry Room in Battery Production.

Gemini Data Loggers (UK) Ltd., 2014. Tinytag Energy Logger. Technisches Handbuch.

KIT 20 Li-ion cell, 2020. . Karlsruhe Institute of Technology, Battery Technology Center.

Pettinger, K.-H., 2019. Telephone Interview: Energy Demand of a Lithium-Ion Battery Cell Production.

Samimi, M.H., Mahari, A., Farahnakian, M.A., Mohseni, H., 2015. The Rogowski Coil Principles and Applications: A Review. IEEE Sens. J. 15, 651–658. https://doi.org/10.1109/JSEN.2014.2362940

Yuan, C., Deng, Y., Li, T., Yang, F., 2017. Manufacturing energy analysis of lithium ion battery pack for electric vehicles. CIRP Ann. 66, 53–56. https://doi.org/10.1016/j.cirp.2017.04.109
